# Late stage melanoma is hallmarked by low NLGN4X expression leading to HIF1A accumulation

**DOI:** 10.1038/s41416-024-02758-9

**Published:** 2024-06-20

**Authors:** David Schörghofer, Laurenz Vock, Madalina A. Mirea, Oliver Eckel, Anna Gschwendtner, Jürgen Neesen, Erika Richtig, Markus Hengstschläger, Mario Mikula

**Affiliations:** 1 Institute of Medical Genetics, Center for Pathobiochemistry and Genetics, Medical University of Vienna, Vienna, 1090 Austria; 2 Department of Dermatology, Medical University of Graz, 8036 Graz, Austria

**Keywords:** Melanoma, Cancer models

## Abstract

**Background:**

Despite ongoing research and recent advances in therapy, metastatic melanoma remains one of the cancers with the worst prognosis. Here we studied the postsynaptic cell adhesion molecule Neuroligin 4X (NLGN4X) and investigated its role in melanoma progression.

**Methods:**

We analysed histologic samples to assess the expression and predictive value of NLGN4X in human melanoma. The oncogenic role of NLGN4X was determined by loss or gain-of-function experiments in vitro as well as by analysis of tumorspheres, which were grafted to human skin organoids derived from pluripotent stem cells. Whole genome expression analysis and validation experiments were performed to clarify the molecular mechanism.

**Results:**

We identified that suppression of NLGN4X down regulated the prefoldin member Von Hippel-Lindau binding protein 1 (VBP1). Moreover, loss of VBP1 was sufficient for accumulation of HIF1A and HIF1A signalling was further shown to be essential for the acquisition of migratory properties in melanoma. We re-established NLGN4X expression in late stage melanoma lines and observed decreased tumour growth after transplantation to human skin organoids generated from pluripotent stem cells. In line, we showed that high amounts of NLGN4X and its target VBP1 in human patient samples had a beneficial prognostic effect on patient survival.

**Conclusion:**

In view of these findings, we propose that decreased amounts of NLGN4X are indicative of a metastatic melanoma phenotype and that loss of NLGN4X provides a novel mechanism for HIF induction.

## Introduction

Melanoma is a type of skin cancer that arises from melanocytes, the pigment producing cells of the skin, and it is responsible for the majority of skin-cancer related deaths [1]. Alarmingly, melanoma shows a continuous rise in incidence rat e of about 5% per year [2]. At the early stages, surgical intervention is able to effectively cure melanoma. However, later stages exhibit a high probability for metastasis leading to early patient death [1]. Despite ongoing research, current melanoma markers fail to reliably predict patient outcome [3]. Therefore, it is of profound importance to identify novel markers and mechanisms involved in melanoma progression.

Melanocytes are suspected to form synaptic contacts with peripheral nervous cells, which may play a role in the regulation of differentiation and melanocyte proliferation [4]. Interestingly, especially glutamate receptors present in the postsynaptic membrane have been linked to the development of melanoma [5–8].

Here we focused on the role of NLGN4X (Neuroligin 4X) during melanoma progression and investigated its association with clinical parameters as well as its functional contribution to melanoma pathology. The neuroligin family comprises five genes, which are all coding for membrane proteins enabling cell to cell adhesion [9, 10]. Despite their crucial function for the maturation and maintenance of synapses [11–13], the role of neuroligins in melanoma development has not been investigated to date.

In the present study, we show that NLGN4X is associated with non-metastatic tumour growth. By modulating NLGN4X, we identified VBP1 (Von Hippel-Lindau Binding Protein 1), a member of the canonical prefoldin complex, as its downstream target. Depletion of VBP1 was sufficient for activation of the oncogenic HIF1A pathway, which drives tumour cell migration. We further showed that re-expression of NLGN4X decreased tumour growth in a novel tumour model based on human foetal skin organoids generated from pluripotent stem cells. This study demonstrates compelling evidence for a link between NLGN4X-controlled neuronal differentiation and its ability to control HIF1A signalling in melanoma. Furthermore, it identifies a novel mechanism of HIF1A induction by downregulation of VBP1.

## Materials and methods

### Cell culture

Human primary melanoma cell lines MCM1, MCM1G, MCM1D and MCM1DLN were generated by Swoboda et al. [14]. WM793B, 1205Lu and WM35 were commercially acquired from ATCC (Manassas, VA). Melanoma cells were screened for mycoplasma contamination once a week and routinely cultured in MIM medium supplemented with 2% FCS (GE Healthcare, Little Chalfront, UK) containing: 80% MCDB153 (Sigma-Aldrich, St. Louis, MO), 20% Leibovitz’s l15 (Mediatech, Tewksbury, MA), 5 µg/ml Insulin, bovine (Sigma-Aldrich), 0.5 ng/ml Epidermal Growth Factor (EGF) (Sigma-Aldrich), 1.68 mM CaCl_2_ (Sigma-Aldrich), 100 IU/ml Penicillin (Sigma-Aldrich), 100 µg/ml Streptomycin (Sigma-Aldrich) and 2 µg/ml Ciprofloxacin (Sigma-Aldrich).

### siRNA transfection

siRNA transfection was carried out using DharmaFECT (Thermo Fisher Scientific, Waltham, MA) according to the manufacturer’s protocol. Before the day of transfection, melanoma cells were passaged in antibiotic-free 2% MIM and transfected as soon as the cells were 60–70% confluent. 20 µM siRNA (GE Healthcare, Little Chalfront UK) was diluted in 1x siRNA buffer (Thermo Fisher Scientific) to a final concentration of 5 µM. 5 µM siRNA was diluted 1:20 in serum free MIM. In a second reaction batch, DharmaFECT (Thermo Fisher Scientific) was diluted 1:50 in serum free MIM. Both reactions were incubated for 5 min at RT. Subsequently, the two reactions were mixed and incubated for 20 min at RT. Thereafter, the mixtures were added to the cells in a ratio of 1:5 in MIM medium to a final siRNA concentration of 25 nM. Cells were incubated for 24 h at 37 °C in 5% CO_2_. Hereafter, medium was replaced with 2% MIM. The following siRNAs were used: NLGN4X siRNA (GE Healthcare), VBP1 siRNA (GE Healthcare). Non-targeting siRNA (GE Healthcare) was used as a control in all experiments.

### Plasmid transfection

NLGN4X plasmid (Addgene, Cambridge, MA) was mixed with the quadruple amount of PEI (Sigma-Aldrich) for 15 min. This mix was added to prior equally seeded cells in Opti-MEM, Reduced Serum Media (Gibco, Thermo Fisher Scientific, Waltham, MA) when they reached 60–70% confluence.

### VBP1 overexpression

Before the day of transfection, melanoma cells were passaged in antibiotic-free 2% MIM and transfected as soon as the cells were 70–80% confluent. On the day of the transfection, the 2% MIM media was replaced with Opti-MEM, Reduced Serum Media and transfection mixes were prepared. These were assembled according to the manufacturer’s protocol using an equimolar mix of guide complex (CRISPR-Cas9 Synthetic tracrRNA : CRISPRmod CRISPRa crRNA Pool Human VBP1, 2.5 μM), 300 ng CRISPRa-EGFP-dCas9-VPR-mRNA (CAS12025) and 7.5 μL Dharmafect Duo (T-2010-01), diluted in 250 μL Opti-MEM. For the conditions receiving siRNA treatment, extra mixes were prepared containing siRNA (20 μM, Horizon) and 7.5 μL Dharmafect Duo diluted in the appropriate quantity of Opti-MEM. The transfection mixes were added to the respective wells and the cells were incubated for 24 h at 37 °C, 5% CO_2_ before media change. After another 24 h recovery in 2% MIM media, cells were lysed using Laemmli buffer supplemented with 1× Protease and Phosphatase Inhibitors, 1 mM PMSF and 0.5 mM EDTA (Thermo Fisher Scientific, Waltham, MA) and sonication.

### Measurement of cell count, metabolic activity and ROS

To measure cell count and LDH, we seeded cells in triplicates in 6 well plates. siRNA transfection was performed directly in the 96 well plates. To measure cell counts of cell cultures, we used the CASY cell counting system (Schärfe System, Reutlingen, Germany). To test the metabolic activity of cells, Alamar Blue assay (Invitrogen, Carlsbad, CA) was carried out as stated in the manufacturer’s protocol. ROS was measured by incubating cells with 20 µM DCFHDA in PBS for 30 min. Subsequently, plates were measured at an excitation wavelength of 488 nm and an emission wavelength of 535 nm every 15 min. Treatment with 10 mM NAC was used as a control.

### Spheroid invasion assay

For the spheroid invasion assay, cells were harvested and resuspended in MIM-methylcellulose (consisting of 20% methylcellulose and of 80% MIM). Thereafter, cells were distributed in drops of 100 µl in a round bottom 96 well plate at 2500 cells/drop and the plate was centrifuged at 300 g for 6 min. Spheroids were incubated at 37 °C (5% CO_2_, 95% humidity) for 96 h. Subsequently, spheroids were harvested and implanted into collagen gels consisting of 2 mg/ml collagen I (Corning, Corning, NY), 1.5% methylcellulose and with NaOH, used for neutralisation. Embedded spheroids were cultivated in 2% MIM and incubated at 37 °C (5% CO_2_, 95% humidity). Images were taken 24 h later and the invasive area was evaluated using Image J (National Institutes of Health, Bethesda, MD).

### Transwell migration assay

For the migration assay, MCM1G and WM793B cells were transfected with NLGN4X and non-targeting siRNA as described. Additionally, subgroups of cells were treated with 5 µM YC-1 (Santa Cruz). 40000 cells in 100 µl serum-free MIM were pipetted into transwells (Sigma-Aldrich) with 8 µm pores placed in 24 well plates filled with 600 µl MIM with 10% serum content. Subsequently, cells were incubated for 6 h (WM793B) or 12 h (MCM1G) at 37 °C and 5% CO_2_. Next, cells were washed with PBS and fixated with 4% formaldehyde for 30 min at room temperature. Subsequently, cells were stained with 0.1% crystal violet (Sigma-Aldrich) for 10 min. For analysis, 5 photographs (middle, top, bottom, right and left) were taken of each transwell and cells were counted with ImageJ.

### Quantitative real-time PCR (qRT-PCR)

Total RNA was isolated by directly adding RNA lysis buffer to the cells. The procedure was performed with peqGold total RNA kit (Peqlab, Erlangen, Germany) according to the manufacturer’s instructions. cDNA was synthesised from 5 µg total RNA using GoScript Reverse Transcription system (Promega, Fitchburg, WI) following manufacturer’s instructions. Quantitative Real-time PCR (SYBR Green) was performed using GoTaq® QPCR Master Mix (Promega), in a StepOnePlus Real-Time PCR Detection System (Thermo Fisher Scientific, Waltham, MA). Cycling conditions: 95 °C for 2 min, 40 cycles at 95 °C for 15 s and 65 °C for 1 min. For each reaction batch, 2 µl of the sample cDNA was mixed with 10 µl GoTaq qPCR Master Mix (Promega), 0.4 µl forward and reverse primer (Eurofins MWG Operon, Luxembourg) and 7.2 µl ddH_2_O. Primer sequences were as followed: NLGN4X: Forward 5′-CTA CTG CTC CCT GGA AAG CCC TAT- 3′, Reverse 5′-TGA ACA ACA AAG GAA GCC ATA GCA- 3′. VBP1: Forward 5′-TCA CGG AAT CCC GGC GGC-3′, Reverse 5′-TGC AGT CTC ATT CCC AGG CTG TTT C-3′. TXNIP: Forward 5′-CCA GCA ATT GGG GGA AAG AAG GC-3′, Reverse 5′-CTC CAA ATC GAG GAA ACC CCT TTG C-3′. HMOX1: Forward 5′-CCG CAG TCA GGC AGA GGG TG-3′, Reverse 5′-GAG CGG GTG TTG AGT GGG GG-3′. β-Actin: Forward 5′-CTA TCC AGG CTG TGC TAT CCC TGT-3′, Reverse 5′-CCT TAA TGT CAC GCA CGA TTT CC-3′. All experiments were done in triplicates. For normalisation, β-Actin was used and relative gene expression was calculated using the comparative Ct method (2^-ΔΔCt^). Reaction batches were done in duplicates.

### Western blotting

For protein isolation, cells were directly lysed in whole cell extraction buffer RIPA (Cell Signalling, Danvers, MA) containing 1 mM PMSF (Sigma-Aldrich, St. Louis, MO) and 1% PIC (Sigma-Aldrich). Samples were centrifuged for 20 min at 16,000 rcf at 4 °C. Subsequently, supernatant was collected and stored at −80 °C. Protein concentration was measured using the BCA Protein Assay Kit (Cell Signalling) according to manufacturer’s instructions. 20–40 µg proteins per sample were denatured at 95 °C for 5 min and loaded onto a TGX Stain-Free^TM^ FastCast^TM^ Acrylamide gel (Bio-Rad, Hercules, CA). Subsequently, the separated proteins were electroblotted onto an LF-PVDF or nitrocellulose membrane. The following primary antibodies were applied for immunodetection as follows: rabbit polyclonal anti-NLGN4X (Sigma-Aldrich, HPA001651, 1:1000, previously used by Davidson et al. [15]), rabbit polyclonal anti-VBP1 (Sigma-Aldrich, HPA023230, 1:1000), mouse monoclonal anti-HIF1A (Santa Cruz, Dallas, TX, sc53546, 1:1000), rabbit monoclonal anti-TXNIP (Abcam, Cambridge, UK, EPR14774, 1:1000), rabbit monoclonal anti-pH2AX (Cell Signalling, 9718, 1:1000), mouse monoclonal anti-HA-Tag (Cell Signalling, 2367, 1:1000). On the following day, membranes were incubated with HRP-linked heavy and light chain antibodies (Anti-Mouse IgG: Thermo Fisher Scientific, Waltham, MA, 31430, 1:5000, Anti-Rabbit IgG: Thermo Fisher Scientific, 31460, 1:5000). Pierce® ECL Western Blotting Substrate (Thermo Fisher Scientific) was used for detection. Total protein was used for normalisation.

### Microarray analysis and GSEA of NLGN4X

Isolated mRNA samples were sent to the Core Facility Genomics (Medical University of Vienna, Vienna, Austria) where whole transcript expression profiling using GeneChip Human Gene 1.0 ST Arrays (Affymetrix, Santa Clara, CA) was conducted. RMA [16] was used for normalisation and limma [17] for inferring differential expression between groups. Expression Console and Transcriptome Analysis Console (Affymetrix) were used for differential gene expression analysis. GSEA was performed using the GSEA tool offered by the Broad Institute (Cambridge, MA). ‘Hallmark Gene Sets’, ‘Curated Gene Sets’, ‘GO Gene Sets’ and ‘Oncogenic Signatures’ were used as gene set universes. Permutation type was set to gene set and analysis was performed with 1000 permutations. The data generated by this study has been deposited in NCBI’s Gene Expression Omnibus [18] and is accessible through GEO Series accession number GSE96632.

### Patient cohort and pathology

With institutional review board approval from the Medical University of Vienna (EK Nr: 1101/2015), tissue slides and microarrays were obtained from the Biobank Graz (Graz, Austria) and US Biomax (Rockville, MD). All samples were formalin-fixed less than 10 min after surgery, paraffin embedded and assembled in tissue arrays as cores of 1.5 mm in diameter or whole tissue sections. Tissue arrays and sections were quality controlled for melanoma tissue, representing different stages of disease progression. Each individual core was assigned to pathohistological characteristics and was reviewed by two board certified pathologists.

### Generation of NLGN4X overexpression lines

Tumour lines were infected (MOI:15) with a lentivirus carrying the fusion construct NLGN4X-mGFP within their expression plasmid (Origene, RC222932L2). Control cell lines were infected (MOI:5) with a lentivirus carrying a mGFP control particle within their expression plasmid ORF (Origene, PS100071V). One week post infection cells were sorted for GFP expression (Sony, MA900 Multi-Application Cell Sorter). To ensure 100% GFP expression, ten individual clones were picked from each line and, after GFP signal testing, pooled again.

### Organoid culture

Skin organoids were generated using human embryonic stem cells WA19 (WiCell CVCL_9780) of early passages (p16–19) after the protocol of Lee et al. [19]. The only modification to the protocol was using 5000 cells per well for the initial seeding.

### Spheroid formation assay

Metastatic tumour lines (controls and re-expressing NLGN4X) were trypsinized and counted and a 10.5 mL cell suspension was prepared for a 96-well plate. This contained 150000 cells (1500 cells per well) in spheroid culture medium (80% vol DMEM with 5% FCS and 1% L-glutamine and 20% methycellulose 33 mM). 100 μL of this suspension was seeded in each well of a TC non-treated, round bottom 96-well plate (SPL Life Sciences). The plate was centrifuged for 10 min at 300 *g* and then placed in an incubator at 37 °C, 5% CO_2_, with no shaking. After 48 h tumour spheroids formed and the spheroid medium was replaced with OMM (organoid maturation medium containing 49.5% vol Advanced DMEM/F12, 49.5% Neurobasal medium, 1% Organoid Matrigel Corning, 100ug/mL Normocyn, 0.1 mM B-Mercaptoethanol, 0.5 × N2 supplement, 0.5 × B27-VitA supplement, 1 × Glutamine).

### Spheroid transplantation

Human skin organoids that were 60 days old were placed on top of 72 h old tumour spheroids in 96 well U-bottom low-attachment plates (SPL Life Sciences). They remained in direct contact for 48 h in OMM at 37 °C, 5% CO_2_, with no shaking. After this time point, when the tumour spheroid attached, they were transferred to 24 well ultra-low attachment plates (Corning CLS3473-24EA) in OMM at 37 °C, 5% CO_2_, shaking (65 rpm) and cultivated for 12 days. Media change was performed every second day. The organoids with attached tumour spheroid are henceforth referred as tumour organoids.

### Immunohistochemistry and Immunofluorescence

Tumour organoids were fixed in 4% paraformaldehyde, washed with 1 × PBS and embedded into 1% agarose molds, processed into paraffin blocks and sectioned. Melanoma tissue arrays and slides, containing paraffin-embedded samples, were melted for 20 min at 60 °C and rehydrated by subsequent incubation in Xylol, Isopropanol, 96% Ethanol, 70% Ethanol and 50% Ethanol. Then, arrays were washed and heated up to 120 °C in a pH 6.0 buffer or a pH 9.0 buffer (Dako, Glostrup, Denmark), depending on the antibody. After cooling to room temperature, samples were incubated with 1% H_2_O_2_ (Sigma-Aldrich, St. Louis, MO) for 10 min. Afterwards, samples were permeabilized with 0.1% TritonX-100 (Sigma-Aldrich) for 5 min. Then, sections were blocked with 2.5% horse sera (Vector Laboratories, Burlingame, CA) for at least 20 min at room temperature. Subsequently, sections were incubated overnight at 4 °C with the primary antibodies directed against anti-NLGN4X (Abcam, EPR13108, 1:2000), rabbit polyclonal anti-VBP1 (Sigma-Aldrich, HPA023230, 1:400) and S100β (Ab52642, Abcam, 1:350, pH 6). On the next day, slides were washed and either secondary biotinylated antibodies (Vector Laboratories), or corresponding fluorescent-labelled antibodies, were added for 45 min at room temperature. After a washing step, sections for immunohistochemistry were incubated for 30 min with Streptavidin-HRP (Leica, Wetzlar, Germany). For detection, arrays were incubated with AEC+ High Sensitivity Substrate Chromogen (Dako). Counterstaining with hematoxylin solution was performed according to Mayer (Carl Roth, Karlsruhe, Germany); arrays were mounted with Aquatex^®^ (Merck Millipore, Billerica, MA).

### Evaluation of immunohistochemical staining

Evaluation of tissue arrays and slides was performed by two independent researchers who were blinded regarding patient details. Immunostaining of the anti-NLGN4X antibody was scored on at least duplicate tissues by using the following arbitrary scale: no staining (0), low staining (1), medium staining (2) and high staining (3).

In addition to the observer-dependent scoring system, we used an computational-supported approach previously established by us [20]. In brief, tissue slides were scanned using a microscopic panoramic slide scanner (3DHistech, Budapest, Hungary). Image J was used to perform colour deconvolution to extract NLGN4X staining from haematoxylin staining and melanin pigment. On the base of these single colour images, automated analysis in ImageJ was used to assign each core an overall staining intensity score by calculating the percentage of stained area and normalising it to the total area of the core.

### Bioinformatics

For mRNA microarray analyses of the MCM1 melanoma cell system data was generated by Swoboda et al. [14], accessible through GEO Series accession number GSE36035. For Kaplan Meier analyses on overall survival time, we analysed mRNA microarray files from Bogunovic et al. [21], accessible through GEO Series accession number GSE19234, and the TCGA consortium [22].

### Statistics

For bar diagrams, standard error of the mean (SEM) and two-tailed *P* values were calculated by performing unpaired (independent) Student’s or Welch’s *t* test in SPSS v21 (IBM, Armonk, NY). Levene’s test with a threshold of 0.05 was performed to choose the appropriate t-test. For multiple comparisons, One-way ANOVA was calculated. Tukey HSD was used for post-hoc analysis. For line diagrams, standard deviation (SD) was calculated in GraphPad Prism 6 (GraphPad Prism Inc., La Jolla, CA). Two-tailed *P* values were calculated by performing unpaired (independent) Student’s or Welch’s t-test in SPSS to compare individual time points and the areas under the curve (AUC). Levene’s test with a threshold of 0.05 was performed to choose the appropriate t-test. For multiple comparisons, One-way ANOVA was calculated.

For scatter plots Pearson correlation analysis was applied to calculate corresponding *R*- and *p* values in SPSS. Kaplan Meier plots analysing effects of NLGN4X expression were generated by classifying the samples into groups of low and high target gene expression according to the median gene expression value (GSE19234) or the NLGN4X staining intensity score (Austrian cohort) in SPSS. Kaplan Meier plot analysing combined effects of NLGN4X and VBP1 expression and corresponding box plots were generated by classifying the samples into groups of low and high risk groups according to the median by the web-based tool SurvExpress [23] and edited in SPSS. To test for the significance of Kaplan Meier analysis, we performed the log-rank (Mantel-Cox) test. Two tailed *P* values for box plots were calculated by performing unpaired (independent) t-test. For analysis of immunohistological staining results, 2 × 2 contingency tables and Fisher’s exact test was applied to determine association of clinicopathological parameters with NLGN4X expression in SPSS.

## Results

### NLGN4X expression decreases with melanoma progression

**Fig. 1:**
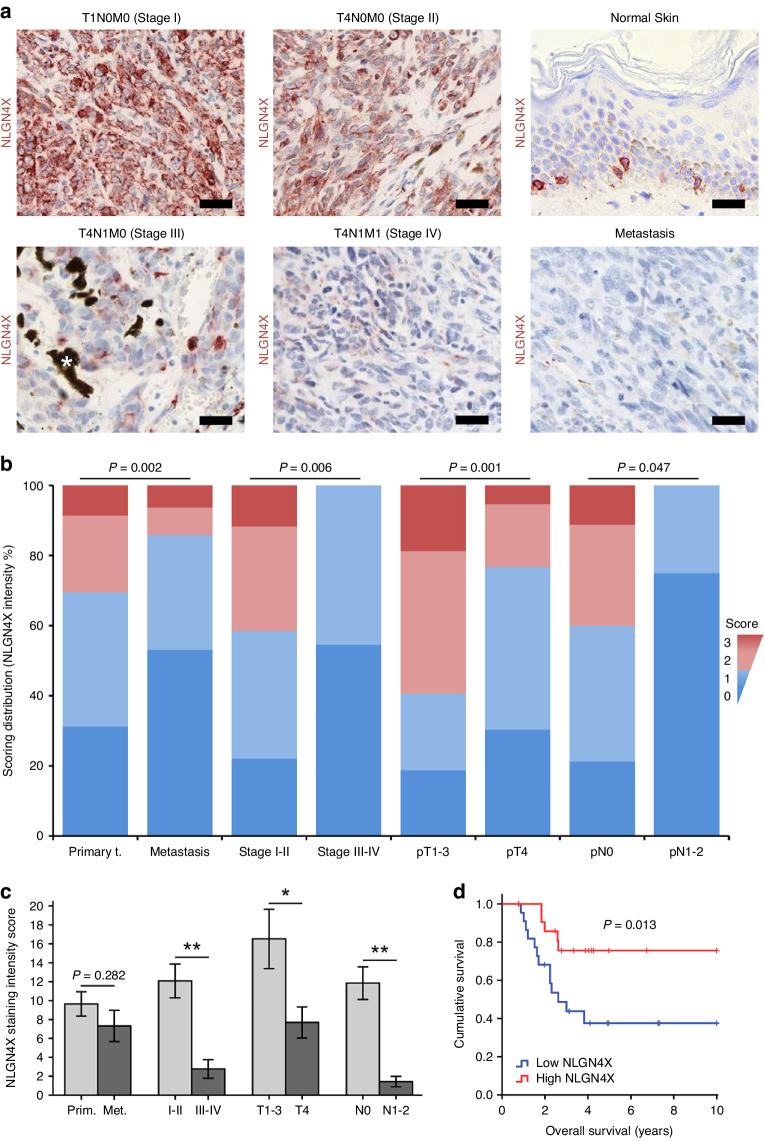
Analysis of melanoma-derived patient samples reveals association of reduced NLGN4X expression with late disease stage and metastasis. **a** Representative tissue sections stained for NLGN4X of stage I, stage II, stage III and stage IV primary melanoma samples, normal skin tissue and melanoma metastasis. * = pigment, bars indicate 30 µm. **b** Overall scoring distribution for NLGN4X staining intensity in different clinicopathological groups. Scores of 0 and 1 were regarded as ‘low’ expression and are shown in blue colours, whereas scores of 2 and 3 were regarded as ‘high’ expression and are shown in red colours. Clinicopathological groups are plotted on the x-axis: primary tumour (t.) and metastasis, pathologic disease stage I–II and III–IV, pathologic tumour stage 1–3 (pT1-3) and 4 (pT4), pathologic lymph node stage 0 (pN0) and 1–2 (pN1-2). Staining was evaluated by grouping samples into high (red) and low (blue) NLGN4X expression and performing Fisher’s Exact Test (*P* values indicated in the graph). **c** Computer-generated NLGN4X intensity scores in primary tumour (Prim.) and metastases (Met.) and primary tumour samples of different pathologic disease stages, pathologic tumour (T) stages and pathologic lymph node (N) stages (see also Fig. S1B-E). Data presented as mean (±SEM), an independent (unpaired) t-test was used for statistical comparisons. **d** Kaplan Meier plot of patients with low and high NLGN4X expression from the dataset GSE19234 (*n* = 22/group). The *P* value of the log-rank test is provided within the graph. * = *P* < 0.05, ** = *P* < 0.001.

To investigate the role of NLGN4X expression in melanoma we acquired clinical melanoma samples from a US patient cohort comprising 249 samples. Tissues were immunohistochemically stained for NLGN4X and analysed for their staining intensity. Pathohistological characteristics of the cohort are listed in Supplementary Table S1. Histochemical analysis frequently revealed strong NLGN4X staining in samples of low stage primary melanoma when compared to samples of high stage primary melanoma (Fig. 1a). Melanocytes in normal human skin exhibited strong NLGN4X staining, while NLGN4X staining was exceptionally weak in individual metastatic samples. Samples were subjected to a scoring system based on staining intensity ranging from 0 (no staining) to 3 (high staining) (Fig. S1A). Scoring distributions according to clinic-pathological characteristics showed that low NLGN4X expression coincided with metastasis (*P* = 0.002), advanced disease stage (I–II vs III–IV, *P* = 0.006), high pT stage (pT1-3 vs pT4, *P* = 0.001) and high pN stage (pN0 vs pN1-2, *P* = 0.047) (Fig. 1b).

To exclude any observer bias, we also performed computer-assisted image analysis. After processing of high resolution images, we could confirm that NLGN4X staining intensity decreased significantly in primary tumours of high pathologic disease stage, high T stage and positive N stage (Fig. 1c, Fig. S1B-D).

To further explore the role of NLGN4X in melanoma patients we analysed the well characterised mRNA microarray file GSE19234 [21]. Kaplan Meier analysis identified significantly prolonged overall survival time for patients with high *NLGN4X* expression as compared to patients with low *NLGN4X* expression (*p* = 0.013) (Fig. 1d). These results establish NLGN4X as a differentiation marker indicative for early stage melanoma.

### Whole genome expression changes after NLGN4X depletion

**Fig. 2:**
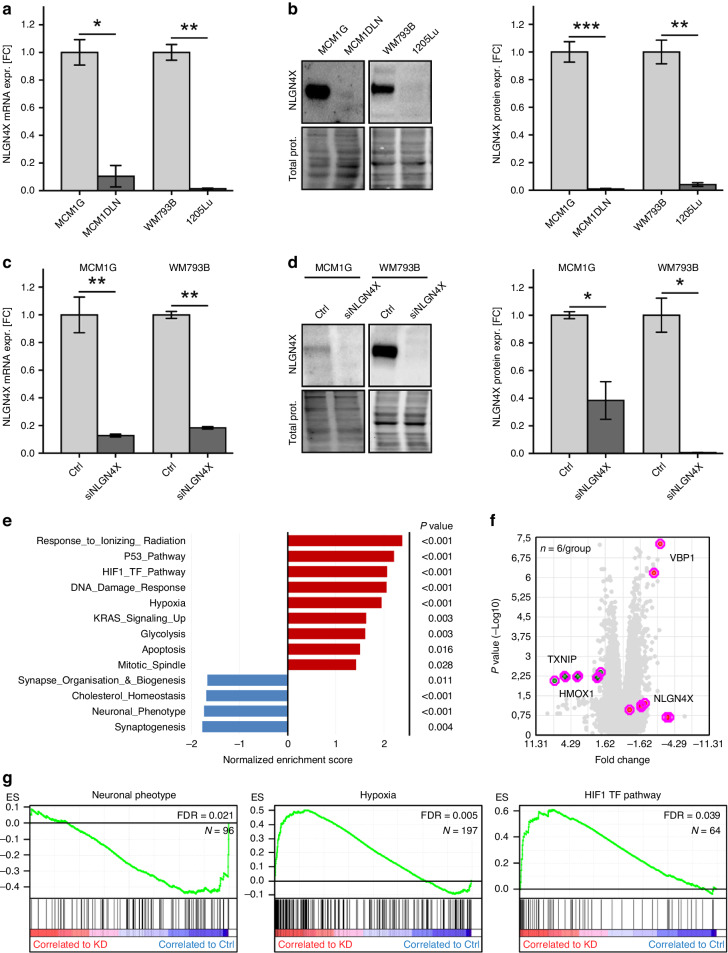
Knockdown of NLGN4X in non-metastatic melanoma cells induces de-differentiation, HIF signalling and DNA damage repair. **a** qPCR data normalised to β-actin comparing NLGN4X mRNA expression of MCM1G to MCM1DLN cells and WM793B to 1205Lu cells (*n* = 3/cell line). **b** Western blots (left) showing NLGN4X and total protein (prot.) loading of indicated cell lines (see also Fig. S2). Corresponding NLGN4X protein expression (right) normalised to total protein (*n* = 3 / cell line). **c** qPCR data normalised to β-actin displaying NLGN4X mRNA expression of MCM1G and WM793B cells treated with non-targeting control (Ctrl) and NLGN4X siRNA (siNLGN4X, *n* = 3/cell line and treatment). **d** Western blots (left) showing NLGN4X and total protein loading of indicated cell lines treated with non-targeting control (Ctrl) and NLGN4X siRNA (siNLGN4X, see also Fig. S3A). Corresponding NLGN4X protein expression (right) normalised to total protein (*n* = 3/cell line and treatment). **e** Log2 mRNA expression (microarray data) of WM793B and MCM1G cells treated with non-targeting control (Ctrl) and NLGN4X siRNA. Right side = genes more than 1.5 fold expressed in control cells, left side = genes more than 1.5 fold expressed in knockdown cells. **f** Major up- and down-regulated Hallmark Gene Sets after NLGN4X knockdown, the nominal *P* value is indicated on the right. **g** Enrichment plots showing the gene sets ‘Neuronal Phenotype’, ‘Hypoxia’ and ‘HIF TF Pathway’ plotted to the enrichment score (ES). The false discovery rate (FDR) and the number of genes in the gene set (N) are given within the graph. Ctrl = control, KD = NLGN4X knockdown. All qPCR and Western blot data presented as mean (±SEM), independent (unpaired) t-test was used for statistical comparisons. Data is shown in fold change (FC) from the mean of non-metastatic (MCM1G, WM793B) samples for cell line comparison or control (Ctrl) samples for knockdown evaluation. kDa kilo Dalton, * = *P* < 0.05, ** = *P* < 0.01, *** = *P* < 0.001.

Patient data on NLGN4X prompted us to further identify downstream targets of this neuronal receptor. We first tested pairs of isogenic melanoma cell lines, which differ in outcome after transplantation to immunocompromised mice, for expression of NLGN4X: MCM1G (non-metastatic) and MCM1DLN (metastatic), as well as, WM793B (non-tumorigenic) and 1205Lu (metastatic) melanoma cell lines. At the mRNA level we detected a significantly higher *NLGN4X* expression in the non-metastatic cell lines MCM1G compared to MCM1DLN and WM793B compared to 1205Lu (Fig. 2a). Furthermore, protein amounts of NLGN4X were significantly higher in non-metastatic cells (Fig. 2b). Next, *NLGN4X* was targeted by a mix of four different siRNAs resulting in significantly reduced NLGN4X amounts in MCM1G and WM793B cells (Fig. 2c, d). To screen for pathways and genes altered by NLGN4X knockdown, we performed whole genome mRNA microarray analysis. Using GSEA [24, 25], we screened our data for enriched gene signatures [26, 27]. NLGN4X knockdown lead to a loss of neuronal associated phenotypes; whereas pathways attributed to DNA damage and HIF1A signalling were up-regulated (Fig. 2e). Specific marker gene expression was visualised in a volcano plot (Fig. 2f). Most importantly, the gene set ‘Neuronal Phenotype’ [28] (*p* < 0.001, FDR = 0.021) was down-regulated and the gene sets ‘Hypoxia’ [27] (*p* < 0.001, FDR = 0.005) and ‘HIF TF Pathway’ [29] (*p* < 0.001, FDR = 0.039) were enriched in the knockdown group (Fig. 2g).

### NLGN4X regulates the HIF1A pathway via VBP1

**Fig. 3:**
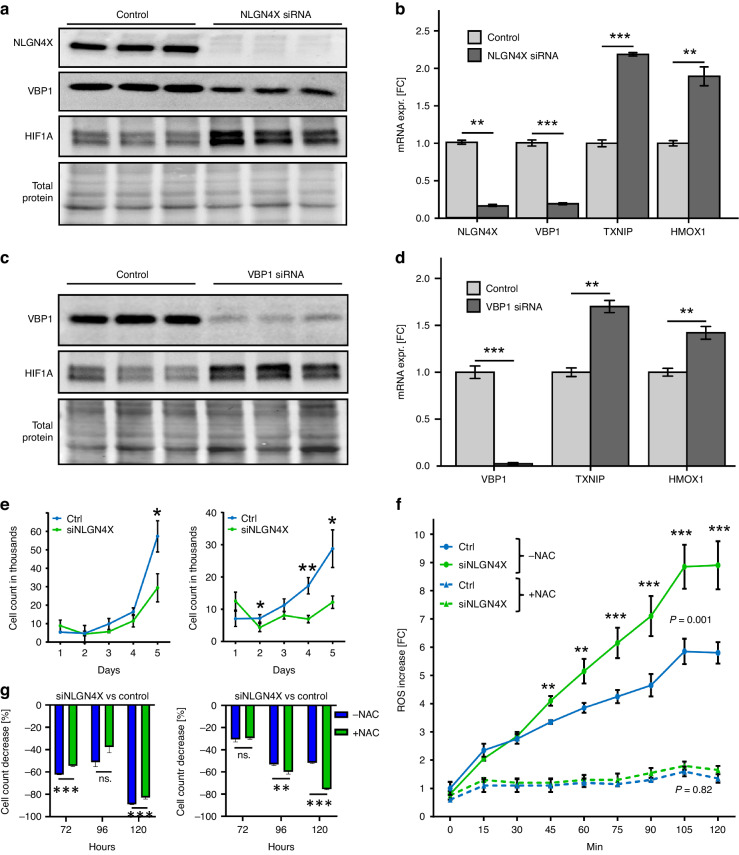
Knockdown of NLGN4X reduces VBP1 and knockdown of VBP1 is sufficient for HIF1A induction. **a** Western blots showing NLGN4X, VBP1, HIF1A and total protein loading of WM793B cells treated with non-targeting control and NLGN4X siRNA (see also Fig. S2). **b** qPCR data normalised to β-actin displaying VBP1, TXNIP and HMOX1 mRNA expression of WM793B cells treated with non-targeting control and NLGN4X siRNA (*n* = 3 / treatment). **c** Western blots showing VBP1, HIF1A and total protein loading of WM793B cells treated with non-targeting control and VBP1 siRNA (see also Fig. S3B). **d** qPCR data normalised to β-actin displaying VBP1, TXNIP and HMOX1 mRNA expression of WM793B cells treated with non-targeting control and VBP1 siRNA (*n* = 3/treatment). **e** Cell count of MCM1G and WM793B cells treated with non-targeting control (Ctrl) and NLGN4X siRNA (siNLGN4X) over a period of 5 days (*n* = 3/cell line and treatment). **f** 793B cells were either control or si NLGN4X treated and ROS was measured by DCHDA fluorescence over time. NAC was used to show reduction of ROS. **g** Cell count from (**e**) was repeated, but cells were treated with 1 µM of NAC or with control solvent. The cell count decrease, after NLGN4X knockdown, is shown for each group. All qPCR and Western blot quantification data presented as mean (±SEM), independent (unpaired) t-or one-way ANOVA and Tukey HSD were used for statistical comparisons. Data is shown in fold change (FC) from the mean of control samples. * = *P* < 0.05, ** = *P* < 0.01, *** = *P* < 0.001. Data presented as mean (±SD), independent (unpaired) t-test was used for statistical comparisons. *P* Values (calculated by independent t-test) for the comparison of the area under the curve are presented within each graph.

We further validated activation of the HIF1A pathway upon NLGN4X knockdown. *VBP1* (VHL binding protein 1) was among the strongest down-regulated genes in both cell models (2.86-fold, *p* < 0.001, FDR = 0.024) (Table S2). VBP1 was shown to closely interact with the tumour suppressor VHL (von Hippel Lindau) [30], a protein playing a crucial role in hypoxic signalling by regulating degradation of HIF1A (hypoxia inducible factor 1A) [31]. Furthermore, TXNIP and HMOX1, two targets of the HIF1A pathway [32–34], were found among the top up-regulated genes in the knockdown group. Protein analysis after NLGN4X knockdown confirmed significant VBP1 down-regulation with simultaneous up-regulation of HIF1A (Fig. 3a and Fig. S2). Additionally, HIF1A targets *TXNIP* and *HMOX1* were also significantly up-regulated (Fig. 3b and Fig. S3A). Since VBP1 was strongly down-regulated in our knockdown samples, we hypothesised that loss of VBP1 was sufficient for HIF1A accumulation. Hence, we targeted *VBP1* in both melanoma cell lines by siRNA. As expected VBP1 protein amounts were significantly reduced (Fig. 3c, Fig. S3B) and importantly, HIF1A accumulated significantly. Consequently, the HIF1A targets *TXNIP* and *HMOX1* showed significant up-regulation in both cell lines (Fig. 3d and Fig. S3C). Increased hypoxia signalling can influence tumour cell proliferation. Hence, we tested for cell number changes after NLGN4X knockdown and we found significant decreases after 5 days of culturing of 793B cells and MCM1G cells (Fig. 3e). To further define the type of cellular stress we measured reactive oxygen species (ROS) generation and identified a strong accumulation in knockout cells, which could be blocked by N-acetyl cysteine (NAC) treatment (Fig. 3f). Interestingly NAC treatment was not able to prevent the cell number decrease after NLGN4X knockdown (Fig. 3g). This indicates that cells where profoundly harmed and that ROS exposure alone is not the reason for reduced proliferation. We further studied the consequences of NLGN4X loss and identified evidence for elevated DNA damage, exemplified by enrichmend of ‘Response to Ionising Radiation’ gene set (Fig. S4A), increased H2AX phosphorylation (Fig. S4B and S4C) aberrant cellular nuclei (Fig. S4D) and finally an accumulation of cells in the G2/M cell cycle phase (Fig. S4E).

### Transient NLGN4X knockdown induces stable HIF1A repression

**Fig. 4:**
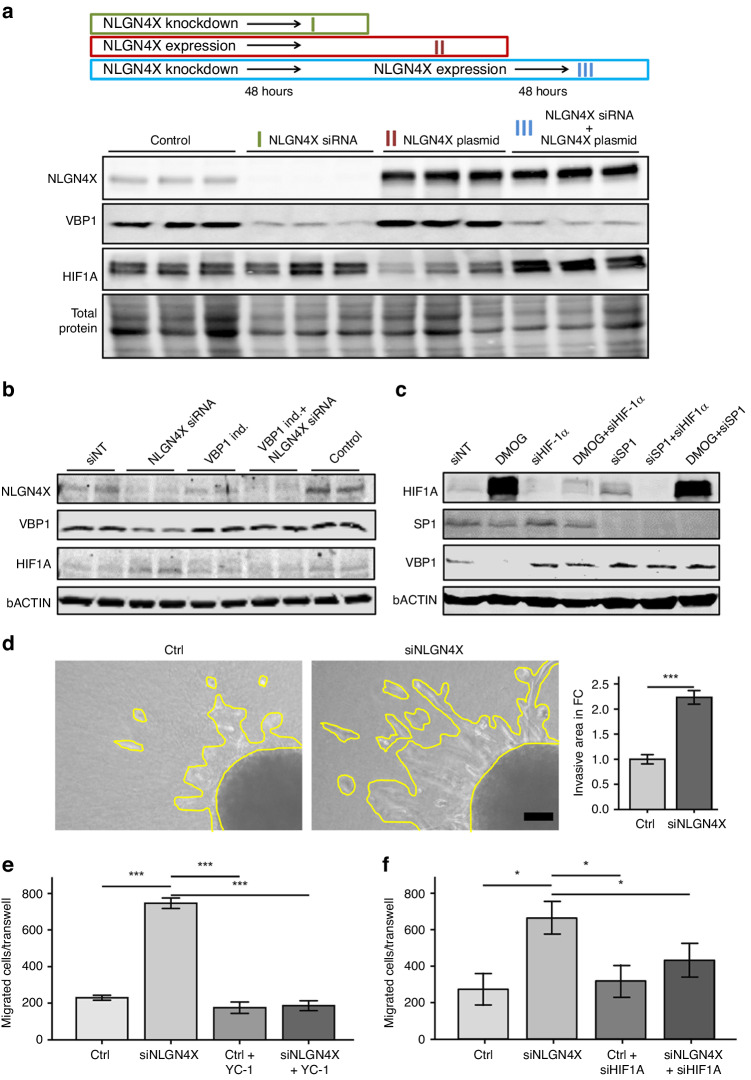
Knockdown of NLGN4X decreases cell proliferation and increases migratory and invasive properties. **a** Western blots showing NLGN4X, HIF1A, VBP1 and total protein loading of WM793B cells treated with non-targeting control siRNA and control plasmid (control), NLGN4X siRNA and control plasmid (NLGN4X siRNA) and non-targeting control siRNA and NLGN4X plasmid. **b** Rescue of NLGN4X knockdown by Cas9-driven induction of VBP1. WM793B cells were either siNLGN4X treated, or VBP1 alone was induced, or both were applied at the same time. HIF1A was detected to evaluate outcome. **c** To analyse VBP1 regulation WM793B cells were harvested 24 h after treatment with DMOG, siHIF1A, siSP1 and combinations thereof. **d** Representative spheroids embedded in collagen gel (left) and corresponding quantification of invasive area (right) of MCM1G cells treated with non-targeting control (Ctrl) and NLGN4X siRNA (siNLGN4X, *n* = 6/treatment). Data presented as mean (± SEM), independent (unpaired) t-test was used for statistical comparisons. Bar indicates 50 µm. **e** Quantification of transwell migration assay of WM793B cells treated with non-targeting control (Ctrl) and NLGN4X siRNA (siNLGN4X) ± YC-1 (*n* = 5/cell line and treatment). **f** Transwell assay was repeated, but instead of YC-1 siHIF1A was applied. Data presented as mean (±SEM), one-way ANOVA and Tukey HSD were used for statistical comparison.

To investigate whether the loss of NLGN4x can be rescued we designed an experiment where we first removed NLGN4X by knockdown and, after 48 h, re-expressed the protein by plasmid transfection (Fig. 4a). Our results showed that, while sole expression of NLGN4X could increase VBP1 amounts as expected, upon prior depletion of NLGN4X, its re-expression did not lead to enhanced VBP1 expression. Additionally, we tested if VBP1 expression rescues HIF1A amounts (Fig. 4b). While loss of NLGN4X induced HIF1A, the concomitant expression of VBP1 was sufficient to lower HIF1A amounts. This indicates that HIF1A regulation by NLGN4X is primarily mediated by VBP1. Since the VBP1 promoter sequence harbours a GC-box element, we next tested if DMOG, a potent HIF1A inducer, could suppress VBP1 expression (Fig. 4c). We found evidence that HIF1A amounts indeed correlated with VBP1 amounts and that expression of the cofactor SP1 was essential for this function.

One of the major consequences of hypoxic signalling in tumours is increased cell migration and invasion. In order to evaluate cell invasion, we generated three-dimensional tumour spheres and cultured them in collagen type I gels. 24 h after spheroid implantation increased invasion was observed in the NLGN4X knockdown group compared to the control group (Fig. 4d). To further investigate the effect of NLGN4X knockdown on migration, we seeded siRNA treated cells into transwells and exposed them for 6 to 12 h to a serum gradient. A significantly higher migratory activity of NLGN4X knockdown cells compared to control cells was observed. Importantly, migration was depending on active HIF1A signalling, since application of YC-1, an inhibitor of HIF-1 activity, or treatment with HIF1A siRNA could block the experimentally induced migration (Figs. 4e, f and S5). To show that HIF1A accumulation occurred without simultaneous protein translation we performed cycloheximide treatment (Fig. S6). Hence, we conclude that loss of NLGN4X stabilised the HIF1A protein leading to an increase of its amount.

### Re-expression of NLGN4X reduces tumour growth in an organoid model

**Fig. 5:**
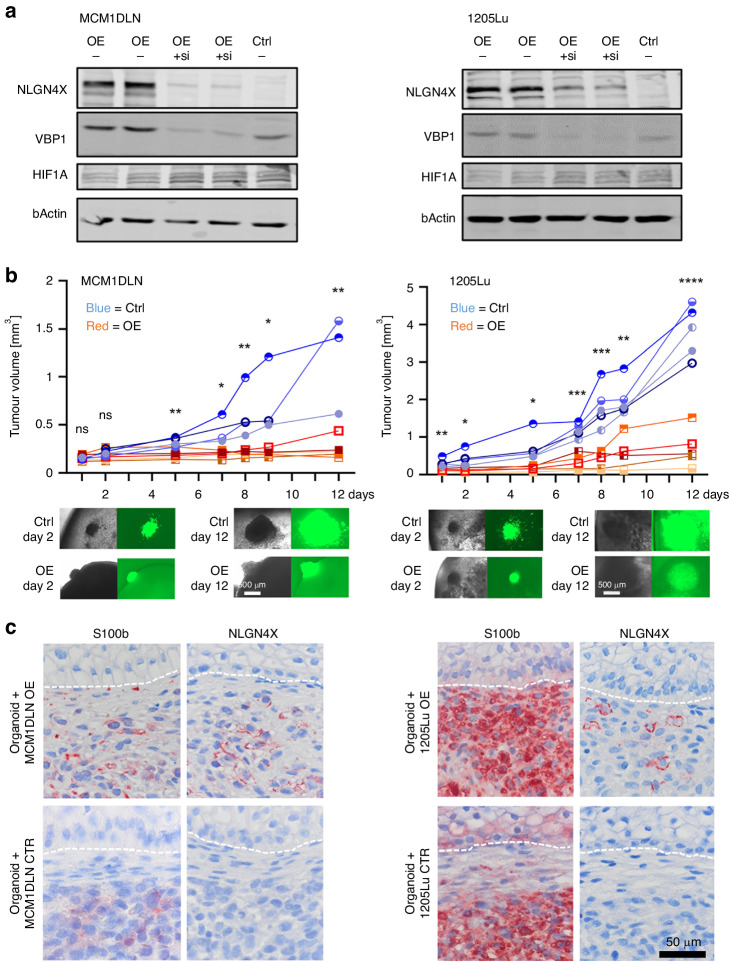
Metastatic tumour cells re-expressing NLGN4X grow slower after grafting onto human skin organoids, compared to their controls. **a** Western blots showing NLGN4X, VBP1 and HIF1A protein expression in metastatic tumour cells lines MCM1DLN and 1205Lu that are stably re-expressing NLGN4X compared to parental cells (Ctrl). siRNA mediated knock down of NLGN4X results in diminished VBP1 expression. **b** Tumour-spheroid volume quantification after grafting onto human skin organoids. Both the control, as well as the NLGN4X re-expressing cell lines, contain a GFP tag that allows for easy quantification of the tumour mass over time. One line represents one individual organoid. Unpaired t-test was used for statistical comparison. Representative images of grafts in organoids at indicated time points are shown in phase contrast and immunofluorescence at the bottom. **c** Immunohistochemistry sections of the human skin organoids 12 days after the tumour spheroid was grafted.

We have shown that late melanoma displays low NLGN4X expression. Hence, we asked how tumour growth changes when the protein is re-expressed in tumour cells. Since NLGN4X is only present in humans, but not in mice, we used a human skin organoid model as the host tissue [19]. By lentiviral infection wildtype NLGN4X was re-expressed in two metastasis-derived melanoma cell lines (Fig. 5a). Interestingly, NLGN4X induced cell lines showed high expression of neuron-associated adhesion markers like PCDHB10 and PCDHB5, which showed down-regulation in NLGN4X knockdown experiments (Fig. S7A–C). Of the NLGN4X re-expressing lines, one showed reduced proliferation and both lines showed reduced invasion of collagen gels compared to control (Fig. S7D, E). Next, human pluripotent stem cells were subjected to a two-stage differentiation protocol, yielding cyst like structures reassembling human foetal skin. Genetically altered melanoma cells were aggregated to form tumour spheroids and grafted onto 60 days old skin organoids. Tumour growth was measured by live cell microscopy over a period of 12 days and, a significant reduction in tumour volume was observed in NLGN4X re-expressing cells compared to GFP expressing control cells (Fig. 5b). Immunhistological staining of samples revealed that not all cells positive for S100b were also positive for NLGN4X (Fig. 5c). Especially 1205Lu cells, which showed the strongest reduction in growth, also showed a higher number of melanoma cells with no detectable NLGN4X expression.

### NLGN4X protein expression indicates progression free survival and correlates with VBP1 amounts

Our cellular models show regulation of VBP1 via NLGN4X. To validate this finding, we acquired an additional 80 clinical melanoma patient samples from an Austrian cohort, which we subjected to immunohistochemical staining for NLGN4X and VBP1. Pathohistological characteristics of the cohort are listed in Table S3.

**Fig. 6:**
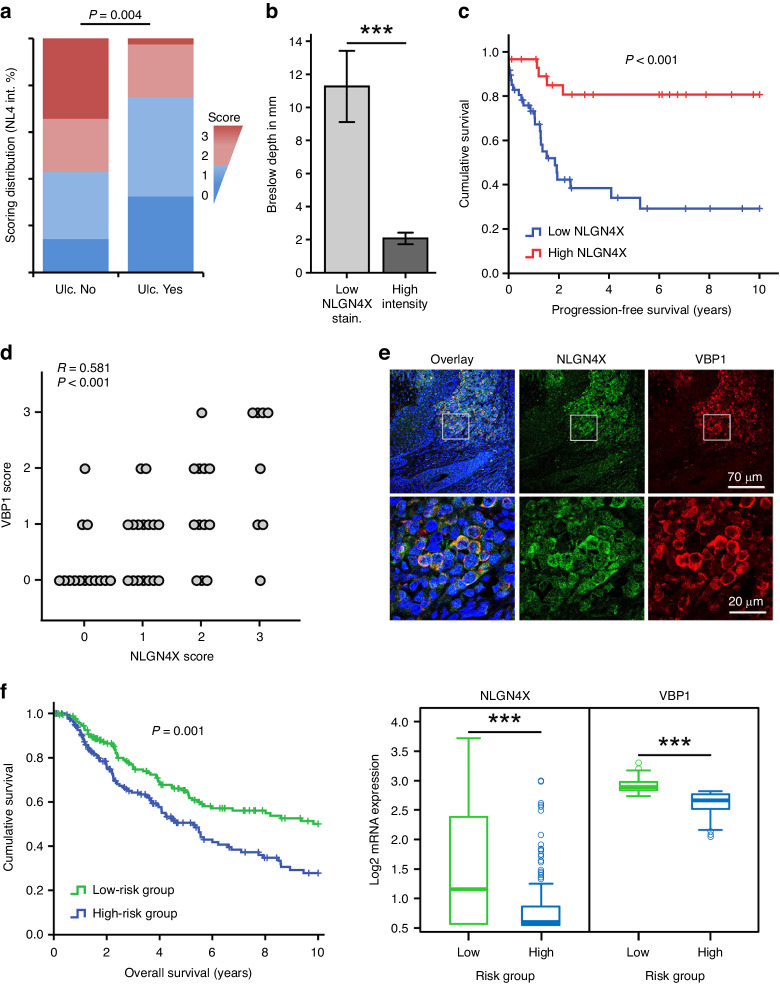
NLGN4X and VBP1 expression correlate in melanoma patients and increase patient survival time. **a** Overall scoring distribution for NLGN4X staining intensity (NL4 int.) in samples distributed according to their ulceration status. Scores of 0 and 1 were regarded as ‘low’ expression and are shown in blue colours, whereas scores of 2 and 3 were regarded as ‘high’ expression and are shown in red colours. Ulceration status is plotted on the x-axis: negative ulceration status (Ulc. No) and positive ulceration status (Ulc. Yes). Staining was evaluated by grouping samples into high (red) and low (blue) NLGN4X expression and performing Fisher’s Exact Test (*P* values indicated in the graph). **b** Breslow Depth in samples of high (*n* = 26) and low (*n* = 42) NLGN4X staining intensity (NLGN4X stain. intensity). Data presented as mean (± SEM), independent (unpaired) t-test was used for statistical comparisons. **c** Kaplan Meier plot of patients with low (*n* = 48) and high (*n* = 30) NLGN4X expression according to antibody staining intensity. The *P* value of the log-rank test is shown. **d** Correlation of NLGN4X and VBP1 histologic staining intensity (*n* = 50). The correlation coefficient (R) and the *P* value of the Pearson correlation analysis is shown. **e** Representative immunofluorescence stain of a human melanoma tissue section for NLGN4X and VBP1 expression. **f** Kaplan Meier plot (left) of patients divided into low (*n* = 168) and high (*n* = 167) risk groups based on their NLGN4X and VBP1 gene expression with corresponding Box plots (right) displaying the expression of the respected genes within the risk groups. The *P* value of the Log Rank test is presented within the survival plot. Independent (unpaired) t-test was used for statistical comparisons of gene expressions. Data was acquired from the TCGA database. *** = *P* < 0.001.

In line with our previous findings, we identified significant association of NLGN4X expression with advanced disease stage (stage I–II vs III–IV, *P* = 0.003), high pT stage (pT1-3 vs pT4, *P* = 0.002), high pN stage (pN0 vs pN1-3, *P* = 0.003) and also with Clark level (*P* = 0.014). Furthermore, we identified ulceration to strongly associate with NLGN4X expression (*P* = 0.004) (Fig. 6a). Additionally, patients with low NLGN4X expression were found to have a significantly higher Breslow depth than patients with high NLGN4X expression (*P* < 0.001) (Fig. 6b). We also analysed progression-free survival, defined as time to distant metastasis and detected patients with high NLGN4X protein expression to have a significantly prolonged progression-free survival time (*P* < 0.001) (Fig. 6c).

For the evaluation of VBP1 stainings, samples were subjected to a scoring system ranging from 0 (no staining) to 3 (high staining) (Fig. S8A). To analyse the correlation between NLGN4X and VBP1 in our melanoma samples, we performed Pearson correlation analysis and identified NLGN4X to significantly correlate with VBP1 (*R* = 0.581, *P* < 0.001) (Fig. 6d). Samples with high correlation were used for immunofluorescence studies or immunohistochemistry and representative samples are shown (Fig. 6e and S8B). To further evaluate the role of NLGN4X and VBP1 in melanoma patients, we used mRNA microarray data from the TCGA consortium [22]. For Kaplan Meier analysis, we stratified patients into low and high risk groups according to their *NLGN4X* and *VBP1* expressions. Patients of the low risk group (high *NLGN4X* and *VBP1* expression) exhibited a significantly prolonged overall survival time compared to patients of the high risk group (low *NLGN4X* and *VBP1* expression) (*p* = 0.001) (Fig. 6f). These results demonstrate that progressive melanoma is hallmarked by the downregulation of both NLGN4X and VBP1.

## Discussion

NLGN4X is known to be mainly present at the axon-dendrite interface and NLGN4X was shown to play a key role in the establishment of cell-to-cell adhesion [13, 35]. Here, we identified NLGN4X as a novel prognostic marker for metastatic melanoma development in human patients. Furthermore, we showed that loss of NLGN4X reduced the neuronal gene signature in melanoma cells and triggered HIF signalling by VBP1 suppression. The NLGN4X-dependent activation of HIF1A was essential for melanoma cell migration and invasion and, re-expression of NLGN4X in metastatic melanoma reduced tumour growth in human skin organoids.

The recent identification of mutations in glutamate receptors has been a major breakthrough in connecting neuronal receptors to melanoma pathology [5–8]. Interestingly, NLGN4X interacts with DLG4 to recruit NMDA receptors [36, 37] and forced expression of NLGN4X in non-neuronal cells was shown to induce synaptogenesis[11, 38]. Our results confirm that the presence of NLGN4X is important for maintaining a neuronal phenotype. Down-regulation of NLGN4X changed neuronal marker expression and induced an invasive phenotype. Interestingly, previous studies have shown that loss of differentiation in melanoma can contribute to worsening of the disease. For example, expression of a primary cilium in melanocytes is a sign of terminal differentiation. Loss of the primary cilium was shown to drive metastatic melanoma formation [39]. These changes are reminiscent of the well-known EMT (Epithelial to Mesenchymal Transition) process, where tumour cells de-differentiate and adopt a metastasis promoting state. Importantly, cells undergoing the EMT process slow down proliferation and show signs of inflammation. For example, the IL6 induced STAT3 pathway is linked to an EMT signature in melanoma and its activation reduced the cell cycle as well as the differentiation associated pigmentation pathway [40]. Also, in this setting de-differentiation was associated with increased metastasis formation. Importantly, here we also identified reduced cell divisions and up-regulation of cell migration, but in our siRNA experiments a full EMT phenotype was not established. This effect was probably due to the use of early melanoma cells, where depletion of NLGN4X mainly induced high cellular stress. Furthermore, it was shown that HIF1A has the potential to antagonise the growth promoting proto-oncogene MYC [41], which could contribute to the observed growth-reduction. Additionally, studies in human breast cancer cell lines showed that siRNA mediated knockdown of NLGN4X lead to induction of apoptosis [42]. Still our analysis of patient material points to an increase in malignancy, upon down-regulation of NLGN4X expression. This might be due to progression of melanoma to a later stage, where additional mutations are acquired to cope with the stress induced by NLGN4X loss whereupon HIF1A activation becomes persistent.

The induction of hypoxic pathways has previously been associated with melanoma progression. Indeed, activation of HIF1A has been shown to play a major role in melanoma initiation, invasion and metastasis [43–47]. When tumour cells acquire the ability to metastasise one of the best-studied metastasis drivers is HIF1A. HIF1A and its transcriptionally regulated target genes are essential for cancer metastasis [48]. HIF1A protein stability is regulated through hydroxylation of proline residues by oxygen-sensitive prolyl hydroxylase domain-containing proteins (PHDs). Furthermore, hydroxylated HIF1A interacts with von Hippel-Lindau protein (VHL), which recruits a multimeric E3 ubiquitinprotein ligase complex to facilitate proteasomal degradation of HIF1A. HIF1A accumulation enables dimerisation with HIF-1β and binding to HIF-response elements of specific genes [49]. VHL is mutated in several cancers, but in melanoma mutations are rare. Instead, regulation of the prefoldin complex, which is required for VHL stability, could be a major pathway for HIF activation [50]. In line, loss of VBP1, a member of the prefoldin complex, has been shown to trigger HIF1A accumulation in B16F10 mouse melanoma cells and in zebrafish [51, 52]. In our study we identified VBP1 as a target of NLGN4X and showed that its reduction also activates the HIF pathway. Mechanistically we showed that SP1 together with HIF1A is necessary to supress VBP1. Importantly GC-box elements were shown to enable binding of SP1 together with MYC and MAX. This interaction activates the respective promoter activity. Conversely, when HIF1A accumulates it displaces MYC and MAX from SP1 leading to promoter suppression. This mechanism has been shown for the genes MSH2 and MSH6 [41] and is likely to also account for VBP1. We observed that transient activation of HIF1A led to sustained VBP1 down-regulation, which could be rescued by NLGN4X in the short term. We suggest that VBP1 down-regulation leads to a feedback-loop which stabilises HIF1A accumulation for a period of time.

To test the effect of NLGN4X re-expression we chose to target metastasis-derived melanoma cells and to graft these cells to a recently developed human skin organoid model [19]. This model has advantages compared to mouse transplantation experiments since it enables human to human cell contact, which is important because NLGN4X is only expressed in the human system. Upon grafting of tumour spheres onto the skin organoid we observed invasion of melanoma cells through the dermis and their reaching of the basal membrane. To our knowledge this is the first time that this pluripotent stem cell-derived organoid has been used to study melanoma growth. Interestingly, although pure clones with re-introduced NLGN4X where used, a strong negative selection occurred and tumour growth was strongly limited. This suggests that once NLGN4X is down-regulated, tumour cells adapt and do not tolerate its re-expression.

## Conclusions

In conclusion, we identified NLGN4X and its target VBP1 as novel melanoma markers down-regulated during melanoma progression. NLGN4X correlated inversely with melanoma stage and patient survival, underlining its potential as a prognostic marker for melanoma. Furthermore, we identified NLGN4X and VBP1 to play fundamental roles in HIF transcription factor stabilisation and tumour cell migration. NLGN4X up-regulation reduced tumour cell growth in a human organoid model indicating its potential role as a tumour suppressor. Hence, low expression of NLGN4X marks late stage melanoma and experimental protocols leading to up-regulation of NLGN4X might represent promising therapeutic options for melanoma patients.

### Supplementary information

Supplementary Material

## Data Availability

The transcriptomic data in this publication have been deposited in NCBI’s Gene Expression Omnibus (GEO Series accession numbers GSE19234). Other data and materials in this paper are available upon reasonable request.
