# Supplementary material for: Late stage melanoma is hallmarked by low NLGN4X expression leading to HIF1A accumulation
Source: Br J Cancer. 2024 Jun 20;131(3):468–80. doi: 10.1038/s41416-024-02758-9 (PMC11300789; doi:10.1038/s41416-024-02758-9)
Supplement: Supplementary file 1 — Supplementary Material [file 41416_2024_2758_MOESM1_ESM.pdf]

Figure S1

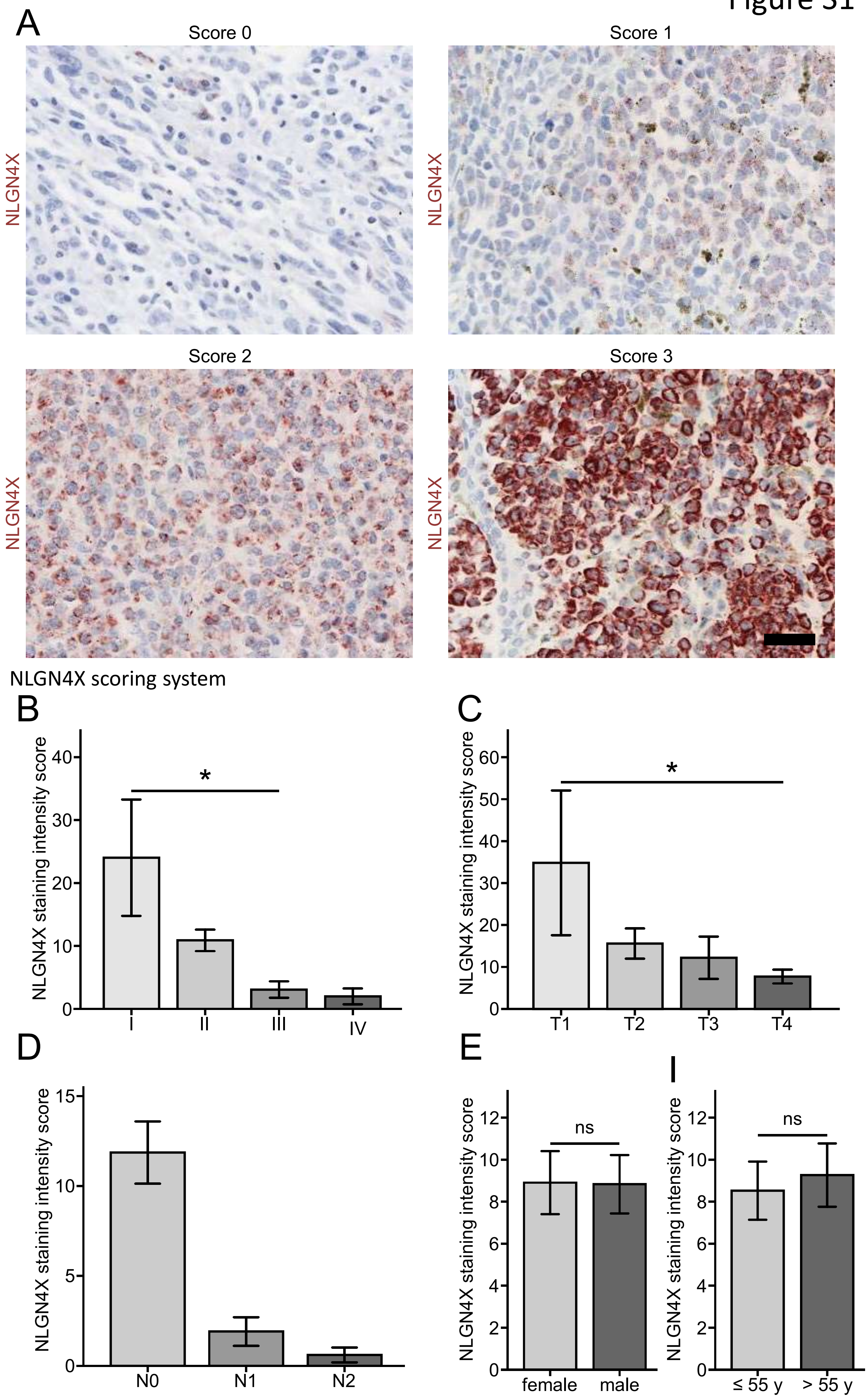
A
Score 0
Score 1
Score 2
Score 3
NLGN4X
NLGN4X scoring system
B
NLGN4X staining intensity score
*
I
II
III
IV
C
*
T1
T2
T3
T4
D
N0
N1
N2
E
ns
female
male
I
≤ 55 y
> 55 y

Figure S2

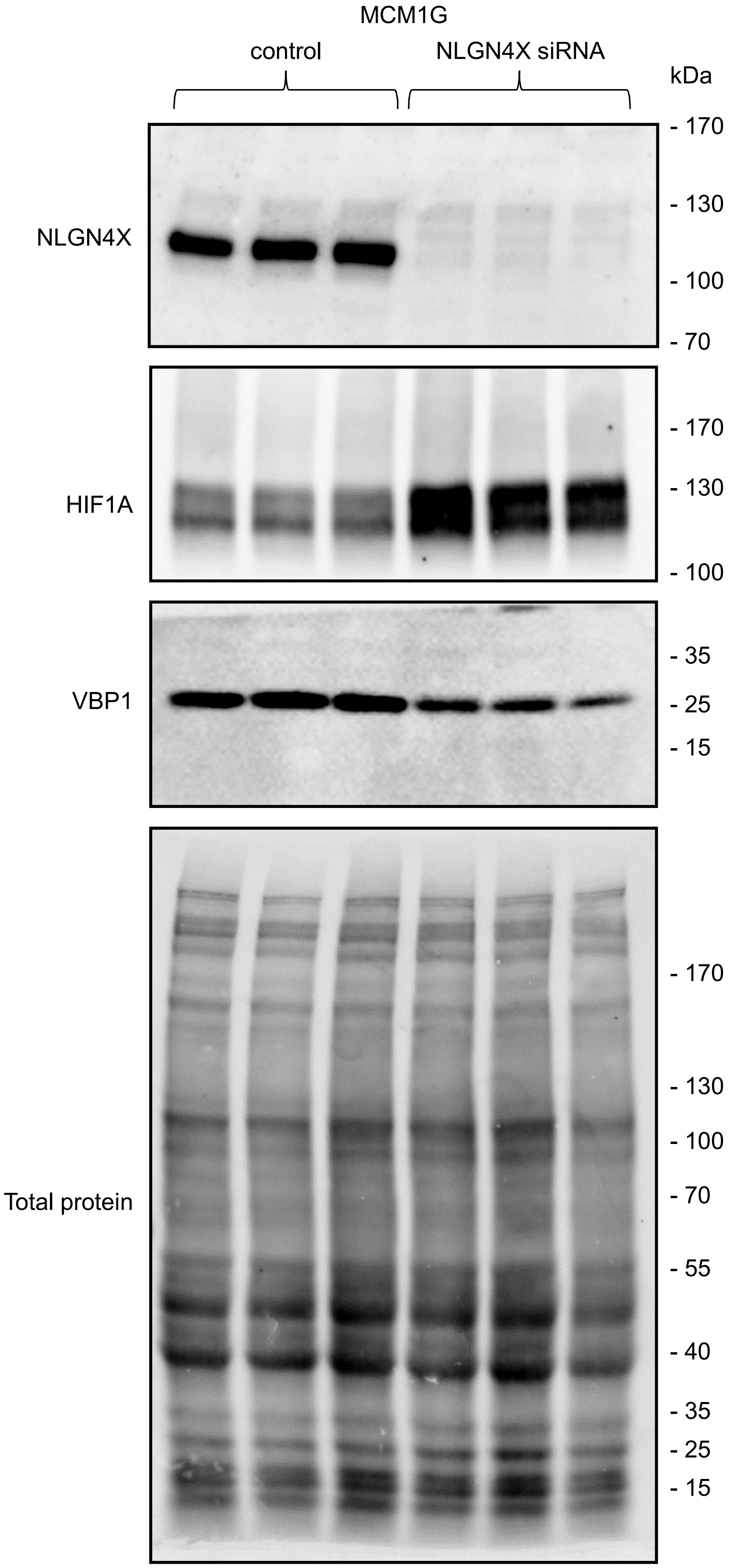

Western blot.

Figure S3

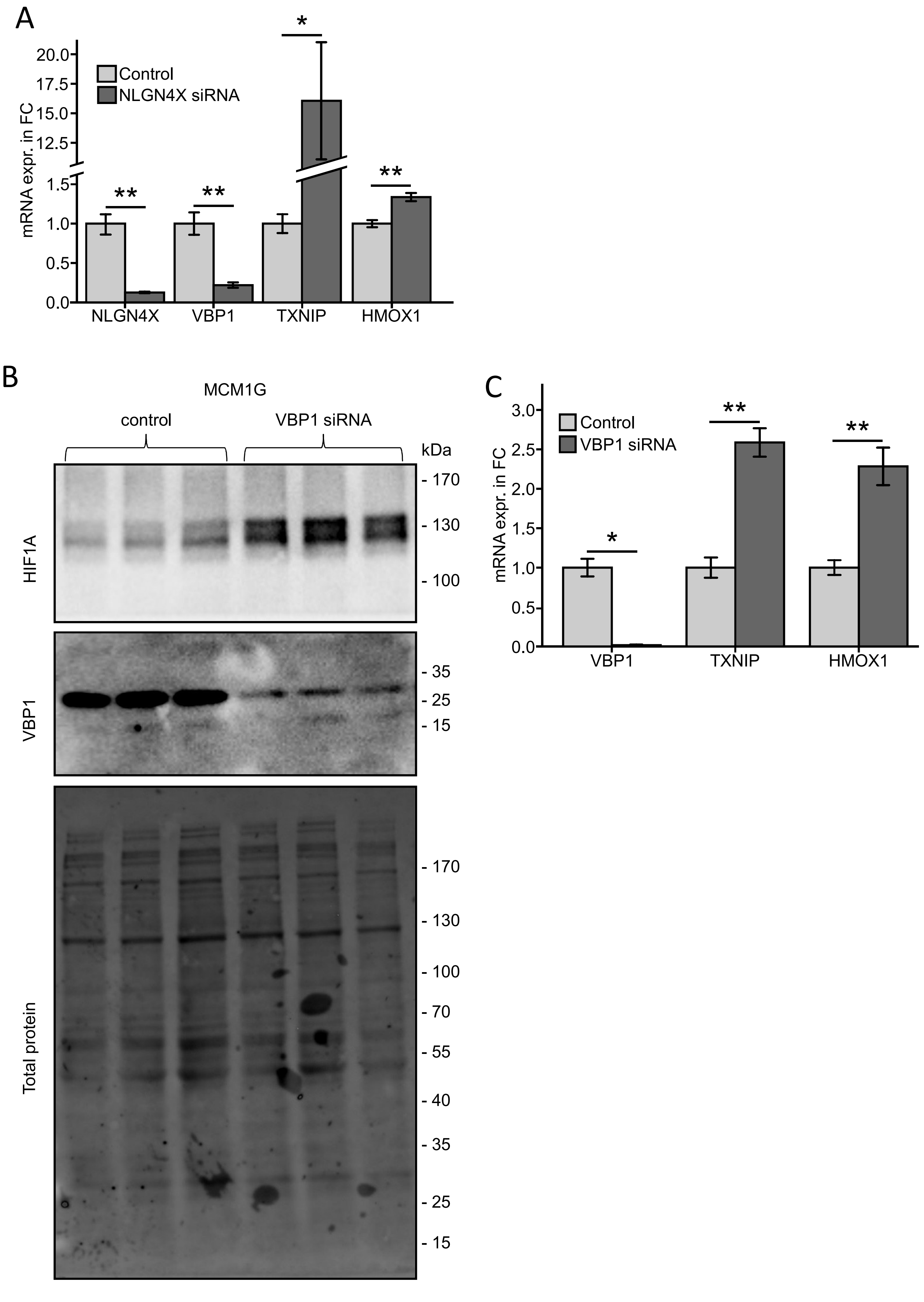

A
Control
NLGN4X siRNA
mRNA expr. in FC
20.0
17.5
15.0
12.5
1.5
1.0
0.5
0.0
**
**
*
**
NLGN4X
VBP1
TXNIP
HMOX1
B
MCM1G
control
VBP1 siRNA
kDa
HIF1A
- 170
- 130
- 100
VBP1
- 35
- 25
- 15
Total protein
- 170
- 130
- 100
- 70
- 55
- 40
- 35
- 25
- 15
C
Control
VBP1 siRNA
mRNA expr. in FC
3.0
2.5
2.0
1.5
1.0
0.5
0.0
*
**
**
VBP1
TXNIP
HMOX1

Figure S4

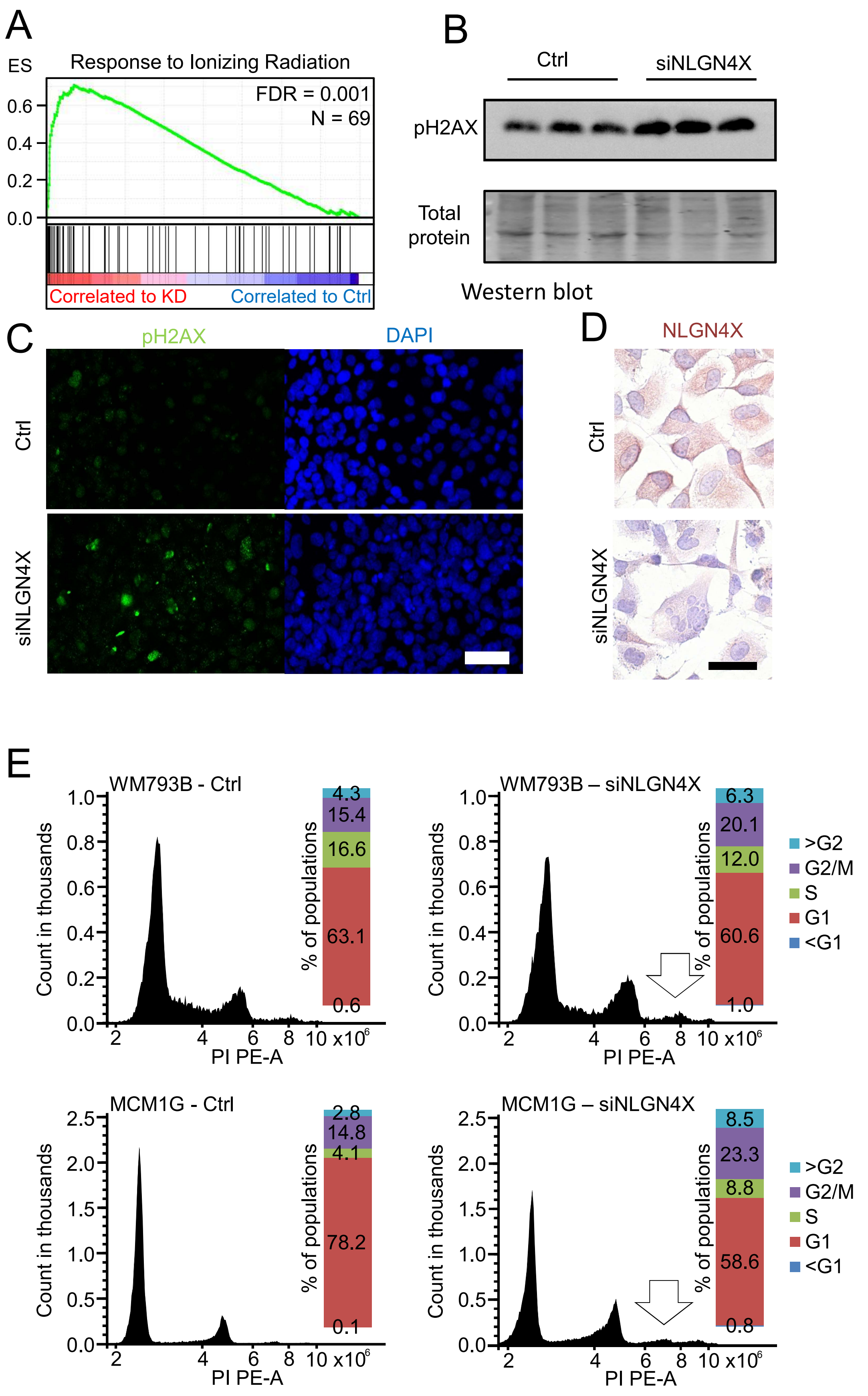
A
ES
Response to Ionizing Radiation
FDR = 0.001
N = 69
0.6
0.4
0.2
0.0
Correlated to KD
Correlated to Ctrl
B
Ctrl
siNLGN4X
pH2AX
Total protein
Western blot
C
pH2AX
DAPI
Ctrl
siNLGN4X
D
NLGN4X
Ctrl
siNLGN4X
E
WM793B - Ctrl
WM793B – siNLGN4X
MCM1G - Ctrl
MCM1G – siNLGN4X
Count in thousands
% of populations
PI PE-A
2 4 6 8 10 x10^6
4.3
15.4
16.6
63.1
0.6
6.3
20.1
12.0
60.6
1.0
2.8
14.8
4.1
78.2
0.1
8.5
23.3
8.8
58.6
0.8
>G2
G2/M
S
G1
<G1

Figure S5

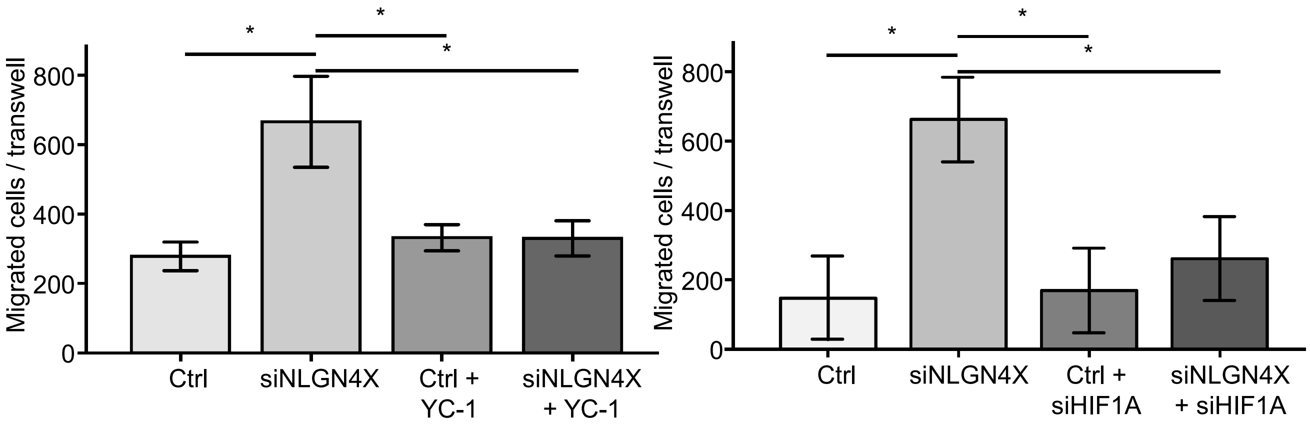
*
*
*
800
600
400
200
0
Migrated cells / transwell
Ctrl
siNLGN4X
Ctrl + YC-1
siNLGN4X + YC-1
*
*
*
800
600
400
200
0
Migrated cells / transwell
Ctrl
siNLGN4X
Ctrl + siHIF1A
siNLGN4X + siHIF1A

Figure S6

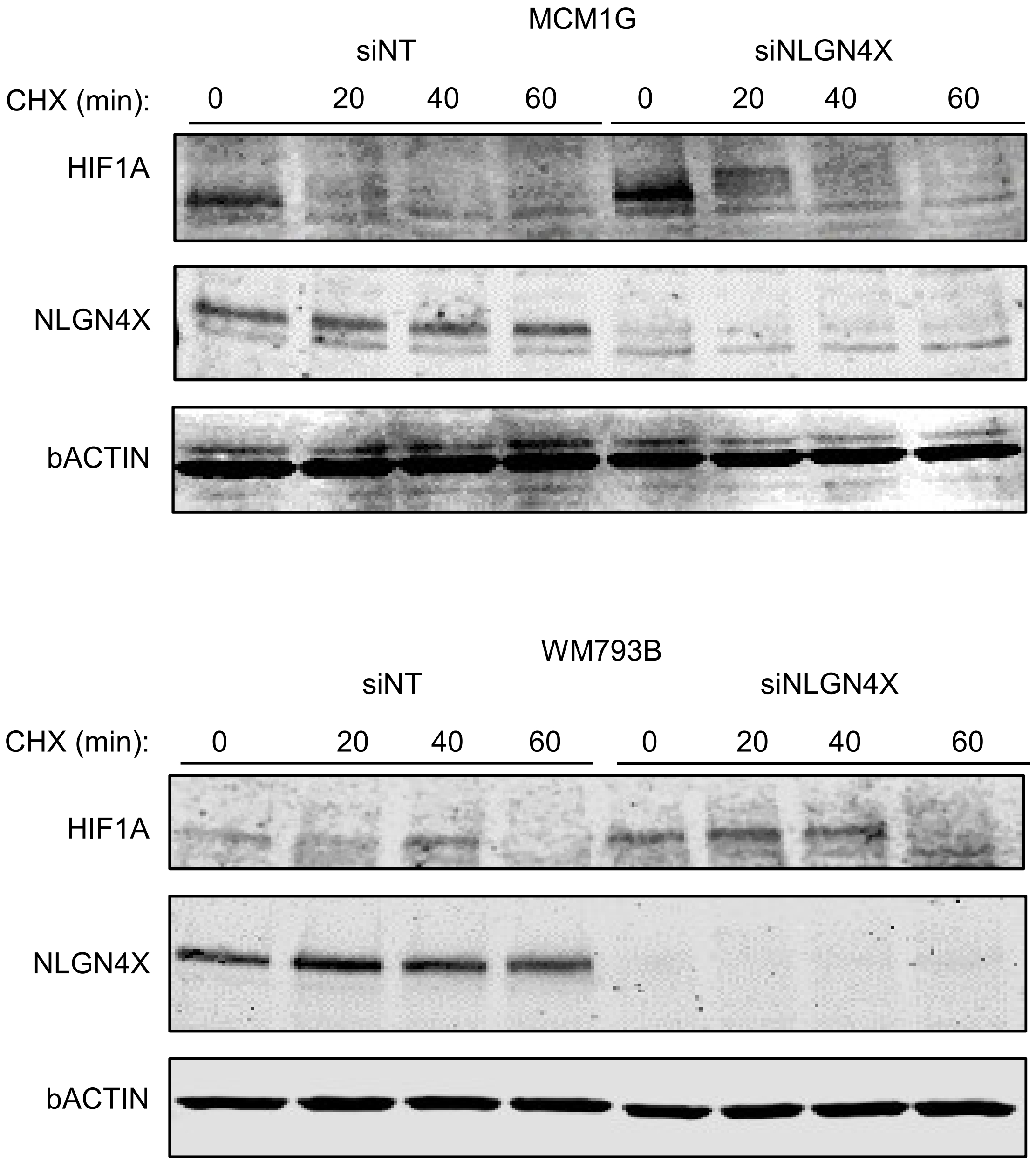
MCM1G
siNT
siNLGN4X
CHX (min): 0 20 40 60 0 20 40 60
HIF1A
NLGN4X
bACTIN
WM793B
siNT
siNLGN4X
CHX (min): 0 20 40 60 0 20 40 60
HIF1A
NLGN4X
bACTIN

Figure S7

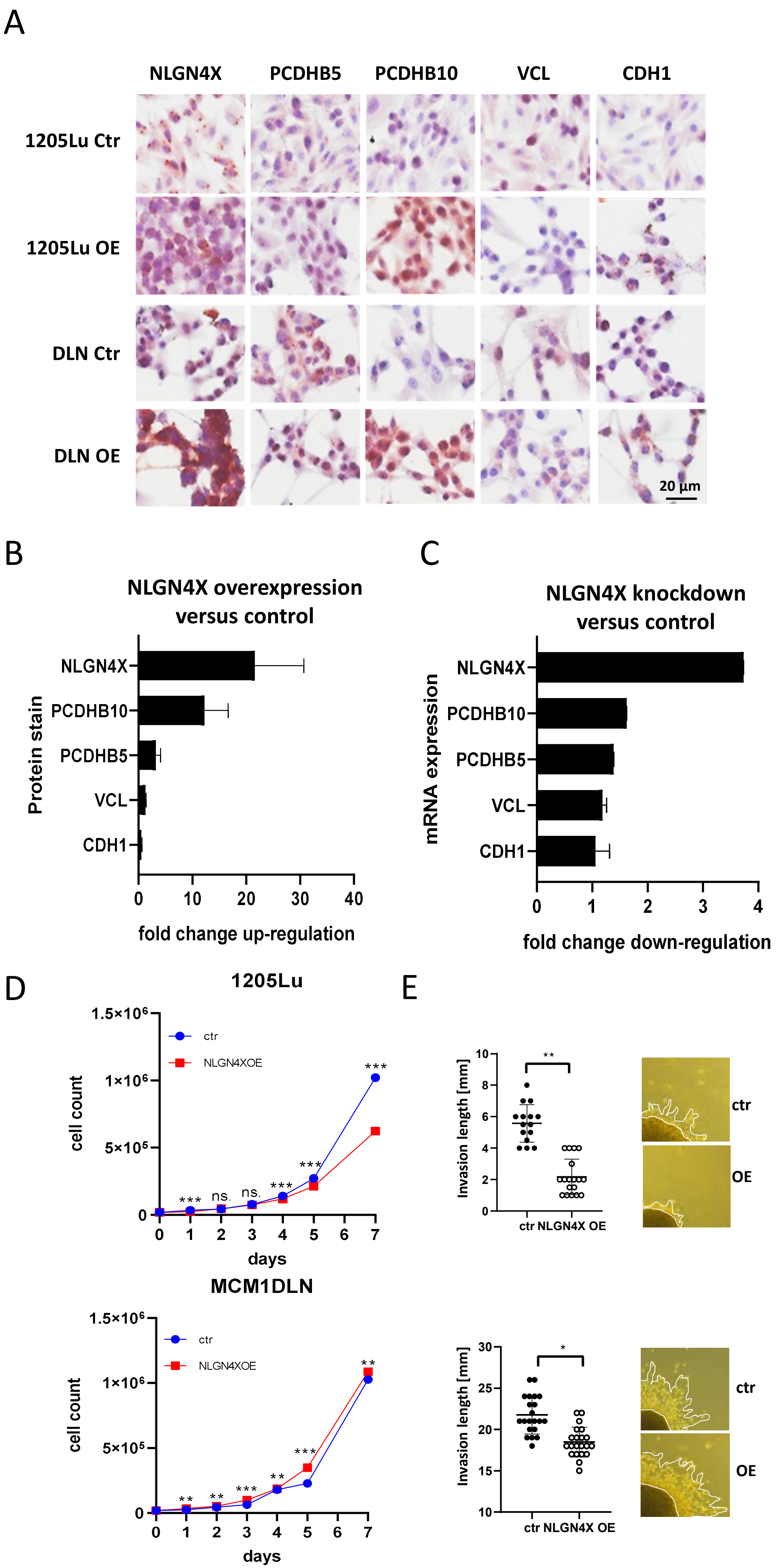
A
NLGN4X
PCDHB5
PCDHB10
VCL
CDH1
1205Lu Ctr
1205Lu OE
DLN Ctr
DLN OE
20 µm
B
NLGN4X overexpression versus control
Protein stain
NLGN4X
PCDHB10
PCDHB5
VCL
CDH1
0
10
20
30
40
fold change up-regulation
C
NLGN4X knockdown versus control
mRNA expression
NLGN4X
PCDHB10
PCDHB5
VCL
CDH1
0
1
2
3
4
fold change down-regulation
D
1205Lu
cell count
1.5×10⁶
1×10⁶
5×10⁵
0
ctr
NLGN4XOE
***
ns.
ns.
***
***
***
0 1 2 3 4 5 6 7
days
MCM1DLN
cell count
1.5×10⁶
1×10⁶
5×10⁵
0
ctr
NLGN4XOE
**
**
***
**
***
**
0 1 2 3 4 5 6 7
days
E
Invasion length [mm]
10
8
6
4
2
0
**
ctr NLGN4X OE
ctr
OE
Invasion length [mm]
30
25
20
15
10
*
ctr NLGN4X OE
ctr
OE

Figure S8

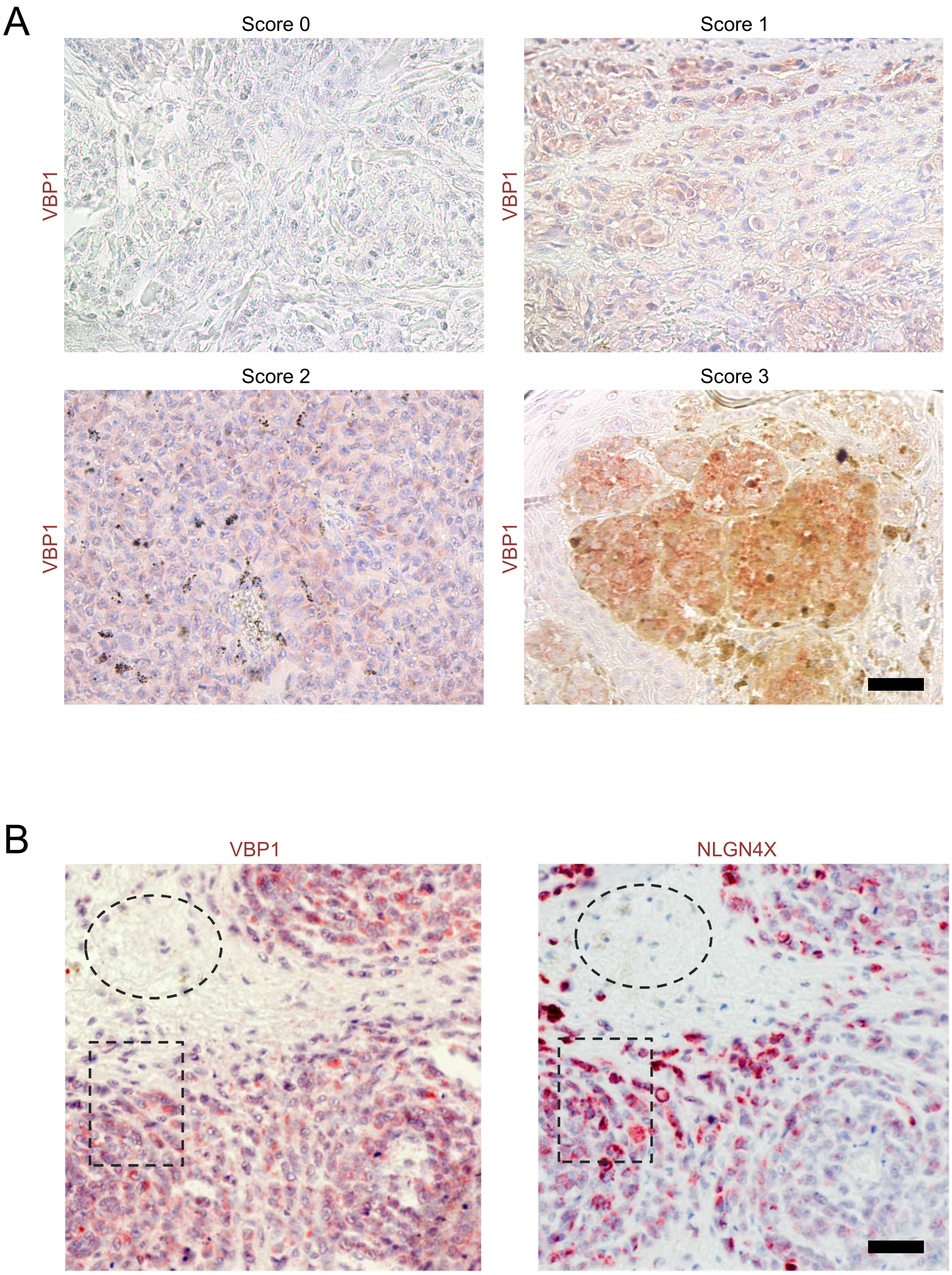

A
Score 0
Score 1
VBP1
VBP1
Score 2
Score 3
VBP1
VBP1
B
VBP1
NLGN4X

Supplementary Figures and Tables

Figure S1: **(A)** Immunohistochemical staining of melanoma tissue for NLGN4X expression is decreased with TNM staging. Panels showing representative melanoma samples scored for NLGN4X staining intensity with 0 for negative , 1 for low , 2 for moderate**)** and 3 for high. Bar indicates 50 µm. **(B)** Computer-generated NLGN4X intensity scores in primary melanoma by pathologic disease stage, **(C)** pathologic T stage (T) and **(D)** pathologic N stage (N). **(E)** Computer-generated NLGN4X intensity scores in melanoma tumor samples (primary tumor and metastasis) by sex and age. Data presented as mean (+/- SEM) **(B-E),** one-way ANOVA and Tukey HSD **(B-D)** and independent (unpaired) t-test **(E)** were used for statistical comparisons. ns = not significant, * = $P < 0.05$.

Figure S2: NLGN4X expression after siNLGN4X treatment in MCM1G cells. Western blots showing NLGN4X, VBP1, HIF1AA and total protein loading of MCM1G cells treated with non-targeting control and NLGN4X siRNA.. kDa = kilo Dalton.

Figure S3: Knockdown of NLGN4X reduces VBP1 and knockdown of VBP1 is sufficient for HIF1AA induction. **(A)** qPCR data normalized to β-actin displaying VBP1, TXNIP and HMOX1 mRNA expression of MCM1G cells treated with non-targeting control and NLGN4X siRNA (n = 3 / treatment). **(B)** Western blots showing VBP1, HIF1AA and total protein loading of MCM1G cells treated with non-targeting control and VBP1 siRNA. **(C)** qPCR data normalized to β-actin displaying VBP1, TXNIP and HMOX1 mRNA expression of MCM1G cells treated with non-targeting control and VBP1 siRNA (n = 3 / treatment).

Figure S4: Effect of NLGN4X loss on DNA damage and cell cycle. **(A)** GSEA analysis showed enrichment of “Response to Ionozing Radiation” geneset in knockdown (KD)

group. **(B)** Western blotting of WM793B cells showed increased H2AX protein phosphorylation after siNLGN4X treatment for 48 hours. **(C)** Immuno fluorescence of cells from (B) showed strong antibody binding (green). **(D)** Immunocytochemistry of cells from (B) showed decreased NLG4X stain in knockdown group and increased number of abnormal nuclei. (**E**) Propidium Iodide FACS of WM793B and MCM1G cells sowed an increased of cells in G2/M cell cycle phase. Arrow indicates polyploidy peaks.

Figure S5: Knockdown of NLGN4X increases migratory properties. Left side, quantification of transwell migration assay of MCM1G cells treated with non-targeting control (Ctrl) and NLGN4X siRNA (siNLGN4X) +/- YC-1 (n = 5 / cell line and treatment). Right side, transwell assay was repeated, but instead of YC-1 siHIF1A was applied. Data presented as mean (+/- SEM), one-way ANOVA and Tukey HSD were used for statistical comparison.

Figure S6: HIF1A accumulation after NLGN4X knockdown. Cells were either control siRNA or siNLGN4X treated and 48 hours later 50 µg/ml cycloheximide was used to block protein translation for 20, 40 and 60 minutes. NLGN4X antibody was used to show knockdown efficiency and beta ACTIN antibody was used as loading control.

Figure S7: Effect of induced NLGN4X expression on melanoma cells. **(A)** Immunocytochemistry of WM1205Lu andMCM1-DLN melanoma cells for NLGN4X, PCDHB5, PCDHB10, VCL and CDH1**. (B)** Quantification of staining intensity from (A). **(C)** mRNA expression was used from GSE96632 and down-regulation of respective genexpression in knockdown cells is shown in fold change. **(D)** Cell proliferation measured by counting in 1205Lu and MCM1DLN control, as well as NLGN4X re-expressing cells over time. **(E)** 1500 cells were aggregated to form a sphere, which was subsequently placed into a collagen I gel. 19 hours later maximum invasion length

per sphere was measured. Data presented as mean (+/- SEM), one-way ANOVA and paired t-test was used for statistical comparisons. * = $P < 0.05$, ** = $P < 0.01$, *** = $P < 0.005$

Figure S8: Analysis of melanoma-derived patient samples reveals association of reduced NLGN4X expression with late disease stage. Overall scoring distribution for VBP1 staining intensity. (A) Scores of 0 and 1 were regarded as "low" expression and are shown in blue colors, whereas scores of 2 and 3 were regarded as "high" expression and are shown in red colors. (B) Representative example for VBP1 and NLGN4X staining in consecutive melanoma sections from an individual patient.

Tables

<u>Table S1: Clinicopathological characteristics of patient samples (US Cohort)</u>

		Count	%
Total		249	100.0
Sex	Female	118	47.4
	Male	131	52.6
Age	≤ 55 years	138	55.4
	> 55 years	111	44.6
Type	Primary tumor	128	51.4
	Metastasis	64	25.7
	Normal tissue	16	6.4
	Non-melanoma cancer	41	16.5

	Count	%	
Total	128	100.0	
Cutaneus	71	55.5	65
Mucinous	35	27.3	2
Eye	13	10.2	13
Soft tissue	9	7.0	8

		Count	%
Total		88	100.0
Stage	I	7	8.0
	II	70	79.5
	III	8	9.1
	IV	3	3.4
T stage	T1	3	3.4
	T2	21	23.9
	T3	8	9.1
	T4	56	63.6
N stage	N0	80	90.9
	N1	5	5.7
	N2	3	3.4
M stage	M0	85	96.6
	M1	3	3.4

Table S2: Top up- and down-regulated genes upon NLGN4X knockdown

Up-regulated	Fold Change	ANOVA p	FDR p
TXNIP	6.77	0.006	0.085
ARRDC4	2.41	0.002	0.051
LIFR	2.26	0.001	0.033
SKIL	2.25	<0.001	0.018
AK4	2.21	0.029	0.186
RHOB	2.15	0.004	0.076
MEST	2.13	0.005	0.084
HMOX1	2.07	0.027	0.18
CLGN	2	0.001	0.049
RBBP6	1.99	0.024	0.172
**Down-regulated**	**Fold Change**	**ANOVA p**	**FDR p**
TMEM8A	-2.15	<0.001	0.028
SBDS	-2.23	0.003	0.066
MIR4746	-2.29	<0.001	0.026
ECE1	-2.64	<0.001	0.018
CEMIP	-2.64	<0.001	0.006
WDR1	-2.65	0.001	0.046
NME4	-2.79	<0.001	0.017
VBP1	-2.86	<0.001	0.024
NLGN4X	-3.45	0.046	0.232
MMP3	-5.68	0.001	0.032

Table S3: Clinicopathological characteristics of patient samples (Austrian Cohort)

		Count	%
Total		80	100.0
Sex	Female	40	50.0
	Male	40	50.0
Age	≤ 65 years	31	38.8
	> 65 years	49	61.3
Type	Primary tumor	80	100.0
Origin	Skin	78	97.5
	Eye	2	2.5
Stage	I	11	13.8
	II	32	40.0
	II	34	42.5
	IV	3	3.8
T stage	T1	11	13.8
	T2	11	13.8
	T3	15	18.8
	T4	40	50.0
	TX	3	3.8
N stage	N0	45	56.3
	N1	15	18.8
	N2	8	10.0
	N3	11	13.8
	NX	1	1.3
M stage	M0	77	96.3
	M1	3	3.8
Ulceration	Yes	40	50.0
	No	35	43.8
	unknown	5	6.3
Clark level	I	0	0.0
	II	4	5.0
	III	9	11.3
	IV	18	22.5
	V	24	30.0
	unknown	25	31.3
Melanoma Progression	Yes	29	36.3
	No	51	63.8
